# Spatial memory deficits after vincristine-induced lesions to the dorsal hippocampus

**DOI:** 10.1371/journal.pone.0231941

**Published:** 2020-04-21

**Authors:** Daniela M. Meléndez, Rebecca E. Nordquist, Louk J. M. J. Vanderschuren, Franz-Josef van der Staay

**Affiliations:** 1 Division of Farm Animal Health, Department of Population Health Sciences, Behaviour and Welfare Group, Faculty of Veterinary Medicine, Utrecht University, Utrecht, The Netherlands; 2 Department of Population Health Sciences, Animals in Science and Society, Faculty of Veterinary Medicine, Utrecht University, Utrecht, The Netherlands; University of Lethbridge, CANADA

## Abstract

Vincristine is a commonly used cytostatic drug for the treatment of leukemia, neuroblastoma and lung cancer, which is known to have neurotoxic properties. The aim of this study was to assess the effects of vincristine, injected directly into the dorsal hippocampus, in spatial memory using the spatial cone field discrimination task. Long Evans rats were trained in the cone field, and after reaching training criterion received bilateral vincristine infusions into the dorsal hippocampus. Vincristine-treated animals presented unilateral or bilateral hippocampal lesions. Animals with bilateral lesions showed lower spatial working and reference memory performance than control animals, but task motivation was unaffected by the lesions. Working and reference memory of animals with unilateral lesions did not differ from animals with bilateral lesions and control animals. In sum, intrahippocampal injection of vincristine caused profound tissue damage in the dorsal hippocampus, associated with substantial cognitive deficits.

## Introduction

Vincristine is a vinca alkaloid obtained from the plant *Catharanthus roseus* commonly used as a chemotherapeutic agent in veterinary and human practice, for the treatment of acute lymphocytic leukemia, acute myeloid leukemia, Hodgkin’s disease, neuroblastoma, and small cell lung cancer [1, 2], among others. Vincristine is a neurotoxic chemotherapeutic agent known to produce sensory and motor, as well as autonomic neuropathies [3]. The toxicity of vincristine is a result of the interruption of the microtubule dynamics, and the induction of mitotic arrest and apoptosis [4–6]. Disturbance of the microtubule formation stops mitosis, directly affecting all rapidly dividing cells, such as cancer cells [7]. A recent study assessing the effect of vincristine on neural tissue concluded that vincristine causes dose-dependent neurotoxicity through the inhibition of the expression of microtubule-related proteins such as tubulin and fribronectin, and the downregulation of the gene matrix metalloproteinase-10 [8].

The present study is a follow-up to a study by Eijkenboom and van der Staay [9], in which cognition deficits were tested using the Morris water maze (MWM) after bilateral injections of vincristine into the dorsal hippocampus in rats. In the present study, injections of vincristine were limited to the dorsal hippocampus because of its involvement in spatial learning [10]. Indeed, there is a wealth of evidence to show that animals with hippocampal lesions have lower performance in spatial tasks such as the cross-shaped maze [11], radial maze [12] and the water maze task [13]. Some of the spatial deficits after hippocampal lesions have been reported to be long-lasting [14].

In the present study, cognition was assessed using the cone field, a complex spatial discrimination task, which can assess both working and reference memory [15–19]. Importantly, hippocampal lesions in laboratory animals have been shown to affect both working and reference memory performance [20–24]. Working memory refers to the process whereby information is temporarily held available for processing [25–27], while reference memory refers to the storage and use of information over a long period of time [28–31]. The sensitivity of the cone field has been well established by testing the effect of compounds such as alcohol, biperiden, scopolamine, haloperidol, d-amphetamine, donepezil and metrifonate [15–19] on working and reference memory. Drugs such as haloperidol impaired reference memory, contrary to d-amphetamine, which impaired working memory [16]. Cognitive disruptors such as scopolamine and MK-801 had a dose dependent effect on working memory and a dose independent effect on reference memory [32], while alcohol and biperiden did not affect either working or reference memory [15,19]. Cognitive enhancers such as the long-acting acetylcholinesterase inhibitor metrifonate improved working memory in healthy rats but had not effect on reference memory [17], while metrifonate was able to antagonize scopolamine working memory deficits. No differences were observed in working and reference memory in animals that received the acetylcholinesterase inhibitor donepezil [18]. The cone field task has the added advantage of providing a test for positively motivated learning behaviour [33], as animals are eager to find food rewards, contrary to the MWM where learning is negatively motivated, as animals attempt to escape from the water tank [34].

The aim of this study was therefore to assess the effects of intrahippocampal injection of vincristine into the dorsal hippocampus, a brain structure implicated in spatial memory, and to assess the sensitivity of the spatial cone field discrimination task in detecting cognitive deficits caused by hippocampal lesions. We predicted that bilateral intra-hippocampal injections of vincristine into the dorsal hippocampus impairs spatial memory.

## Materials and methods

The study was approved by the Animal Ethics Committee of Utrecht University, The Netherlands (DEC 2013.I.04.042), and was conducted in accordance with Dutch laws (Wet op de Dierproeven, 1996) and the EU directive 86/609/EEC.

### Animals and housing

Twenty-four male six-week-old Long Evans rats (RjOrl:LE) were purchased from Janvier Labs (Saint Berthevin, France) and used in an experiment that lasted 6 weeks. Animals were housed in pairs in standard Makrolon type IV cages with sawdust bedding (Abedd, Vienna, Austria), tissue paper and a shelter as enrichment. The animals were housed under controlled climate conditions (21°C ± 1°C, 50–65% humidity), with a radio playing constantly as background noise. The night/day cycle was reversed (lights off from 7:00–19:00) with red light on during the dark phase. Food (CRM, Expanded, Special Diet Services, Witham, United Kingdom) and fresh tap water were available *ad libitum* until and during the first week of habituation. All animals were handled and tested in the same room where they were housed.

### Apparatus

**Fig 1:**
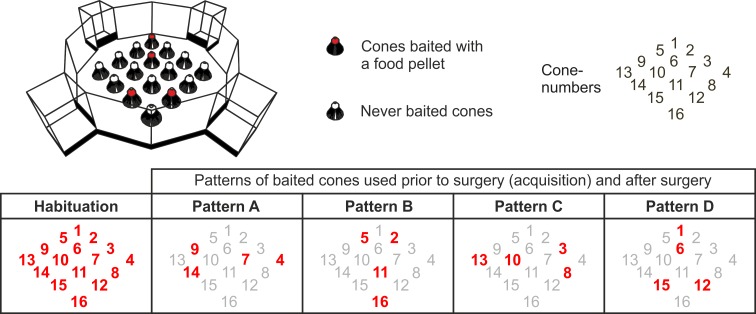
The conefield spatial discrimination apparatus. The pattern of baited cones used during the habituation sessions, and during the training and testing phase (A, B, C and D) are depicted. Cone numbers printed in bold and red indicate that they contained bait (slightly modified from Fig 2 in Bouger & van der Staay, 2005 [32]; reproduced with permission).

The cone field is a dodecagonal open field with a stainless steel floor surrounded by 45 cm high poly vinyl chloride (PVC) transparent walls (Fig 1). The testing arena can be accessed through 4 different starting boxes, which have pneumatic hydraulic doors that are operated through a computer. The arena contains 16 cones, each of 13.5 cm height arranged in a 4 x 4 square, with a food cup inside the top of each cone. A visit to a cone is operationally defined as a leaning response against the collar at the top of a cone.

The food cups of cones were baited with a 45 mg precision sugar pellet (BioServ dustless precision pellets, Frenchtown, NJ). Leaning against the top of a cone was registered as cone visit by activating a capacitive touch sensor; infrared photocells in the food cups detected reward collection. Cones number 4, 7, 9 and 14 were baited (pattern A), or their 90°, 180°, or 270° rotation patterns (B, C, and D) were used during the experiment (see Fig 1. for details). Data were sent to the connected computer and the software randomly selected the start-box for each trial [19]. To mask odor cues all the cones were baited with two additional-45 mg sugar pellets underneath the food cup that were inaccessible for the rat. Extra maze cues consisted of: shelves with the cages housing the rats, a door and a desk with a laptop where the experimenter was always located during a trial. Data of cone visits, food cup visits, as well as trial duration were collected via a computer running the operating system Microsoft Windows 7.

### Habituation and training

After arrival, the animals were habituated during a period of 1 week, in which they were handled by the experimenter once per day. *Ad-libitum* food as well as fresh tap water was provided during this period. The weight of the animals and the weight of the food per cage were recorded daily, in order to calculate daily food intake.

During the second week, the animals were food deprived to approximately 85% of their free feeding weight by feeding 5 g food/100 g of body weight. This was done to increase motivation to search for the food rewards. The feeding schedule resulted in stable weights throughout the experiment. Animals were trained from Monday to Friday and were fed *ad-libitum* during the weekend. On Sunday afternoon, all food was removed again from the food trays. During habituation trials, all cones were baited and some 45 mg sugar pellets (BioServ dustless precision pellets, Frenchtown, NJ) were scattered on the floor of the apparatus. The trial ended after all the sugar pellets from the cones were found, or after 10 minutes, whichever event occurred first. Animals were habituated to the cone field during 7 sessions on 6 consecutive working days. On the last day of habituation, the rats received two sessions, one in the morning and one in the afternoon to ensure that all animals searched for the food reward in all the 16 cones.

After habituation each rat was allocated to a fixed pattern out of the 4 configurations (A, B, C or D, see Fig 1) each containing 4 baited cones [32].

During acquisition, the rats were trained for 60 trials (days 1 and 2: 2 successive trials per day; day 3 and 4: 3 successive trials per day; day 5 to 7: 5 successive trials per day; day 8: 10 trials (5 successive trials in the morning and 5 in the afternoon); days 9 to 13: 5 successive trials per day). After surgery, the rats were tested for 8 days with 5 successive trials per day. A trial started once the animal was placed in the starting box and the door was opened, and ended once the animal found the 4 sugar pellets or after a period of 3 minutes, whichever occurred first. The starting position as well as the order in which the animals were tested was randomized.

Outcomes measured [35] included:

1. Working memory (WM) ratio, defined as the number of rewarded visits divided by the number of visits to the baited set of cones. This ratio measure reflects the ability of the rats to avoid re-visits to cones of the baited set of cones during a trial.
2. Reference Memory (RM) ratio, defined as the number of visits and revisits to the baited cones divided by the number of visits to all the cones. This ratio measure provides an index for the ability of rats to discriminate between baited and unbaited cones.
3. Trial duration, defined as the time (s) elapsed between the beginning (opening the door of the start box) and the end of a trial (finding the last of four baits, or 3 minutes, whichever event occurred first).
4. First visit latency, defined as the time (s) elapsed between the start of the trial and the first cone visit.
5. Inter-visit interval, defined as the average time (s) between cone visits.

### Surgery

After completion of the 60^th^ training trial, animals were randomly allocated to a treatment condition: a group receiving bilateral injections of vincristine into the dorsal hippocampus, or the control group in which a physiological saline solution (0.90% w/v of NaCl) instead of vincristine was injected. In the previous study by Eijkenboom and van der Staay [9], rats injected with a volume of 1.0 or 2.0 μl at a concentration of 0.55 mg/ml of vincristine presented disrupted acquisition in the MWM, however, lesions caused by 2.0 μl extended to the cortical and subcortical regions. This led to a second experiment using 1 μl of vincristine at a concentration of 0.55, 0.18 and 0.06 mg/ml. Although a dose of 0.18 mg/ml did not affect spatial learning in the MWM, this was selected as the dose of choice because lesions were mainly restricted to the hippocampus, contrary to the lesions observed with 0.55 mg/ml which also affected cortical areas.

Due to problems with the initial anesthesia protocol using i.p. fentanyl (0.25 mg/kg; intraperitoneally, i.p.) and dexmedetomidine (0.15 mg/kg, i.p.), the following anaesthesia protocol was used instead: ketamine (75mg/kg; intramuscularly, i.m.) and dexmedetomidine (0.07mg/kg; subcutaneously, s.c.). After loss of the pedal reflex, animals were transported to the surgery room where the head was shaved, eye ointment was applied to prevent drying of the cornea and 8 ml of saline solution was injected s.c. (4 ml on each side of the body) to prevent dehydration. Subsequently, animals were intubated to maintain anaesthesia with isoflurane in 100% oxygen. Capnography was used to monitor the animals throughout anaesthesia (LifeSense®VET Portable Capnography and Pulse Oximetry Monitor) measuring end-tidal carbon dioxide (EtCO2), fractional inspired CO2 (FiCO2), oxygen saturation (SpO2) and respiration rate.

The head area was sterilised done with Betadine 100 mg/ml. A rectal thermometer was used to monitor body temperature while animals were positioned in a stereotactic apparatus (David Kopf Instruments, Tujunga, CA, USA). The scalp was incised, retracted with Backhaus towel clamps and the head position was adjusted to place bregma and lambda in the same horizontal plane. Prior to removal of connective tissue from the periosteum 3 mg/kg of the local anesthetic lidocaine was applied to the area. Small holes were drilled bilaterally into the skull for the entry of the needles (30G, 12mm) into the dorsal hippocampus: AP: -3.1mm, ML: 2.0mm and DV: 3.8mm from bregma. Bilateral injections of 1 μl of vincristine at a concentration of 0.18 mg/ml (i.e., 0.18 μg/hemisphere) were applied to the treatment group, while the control group received 1 μl of saline solution. Injections were done with a 10 μl Hamilton syringe (Hamilton, Reno, NV) driven by a Harvard peristaltic pump at an infusion speed of 0.5 μl/min. Movement of a bubble in the tubing was used to confirm the injection volume. After infusion, the animals were kept in the stereotactic apparatus for 2 minutes before the needles were slowly retracted.

Following surgery, the incision was closed with a vicryl suture in a continuous pattern and anaesthesia was antagonized with atipamezol (0.6 mg/kg) administered i.p.. After surgery, the animals were transported back to the housing room and were placed in individual Eurostandard Type IV S filter top cages (480 × 375 × 210 mm, floor area1500 cm^2^; Tecniplast, Milan, Italy) on top of a heating pad. Postoperative analgesia consisted of buprenorphine (0.05 mg/kg) at 12-hour intervals for 3 days after surgery and meloxicam (0.2 mg/kg) at 24-hour interval for 2 days after surgery.

To ensure adequate healing of the wound, rats stayed housed individually in Eurostandard Type III H filter top cages (425 × 266 × 185 mm, floor area 800 cm^2^) (Tecniplast, Milan. Italy) after surgery for a period of one week. Then, animals were reunited with their original partner. Due to the loss of animals during anaesthesia, two pairs had to be rehoused with an unfamiliar rat from their same treatment group. One week after surgery, a rat from the vincristine group presented a convulsion and was hyper-reactive when handled. Two days after the convulsion, this animal was euthanized, because a humane endpoint, as defined in the approved study protocol, had been reached.

### Histology

Histological verification of the injection site was performed after the completion of the experiment. The animals were decapitated and the brains were rapidly removed and frozen in -80°C 2-methylbutane and subsequently stored at -80°C before further processing. Coronal sections (8 μm) were cut and mounted on Menzel SuperFrost Plus slides (Menzel GmbH & Co, Braunschweig, Germany). Tissue was stained with Cresyl Violet, washed with alcohol 100% and fixated with Xylol. Sections were embedded with Entellan® new (© Merck, Germany) and coverslipped.

**Fig 2:**
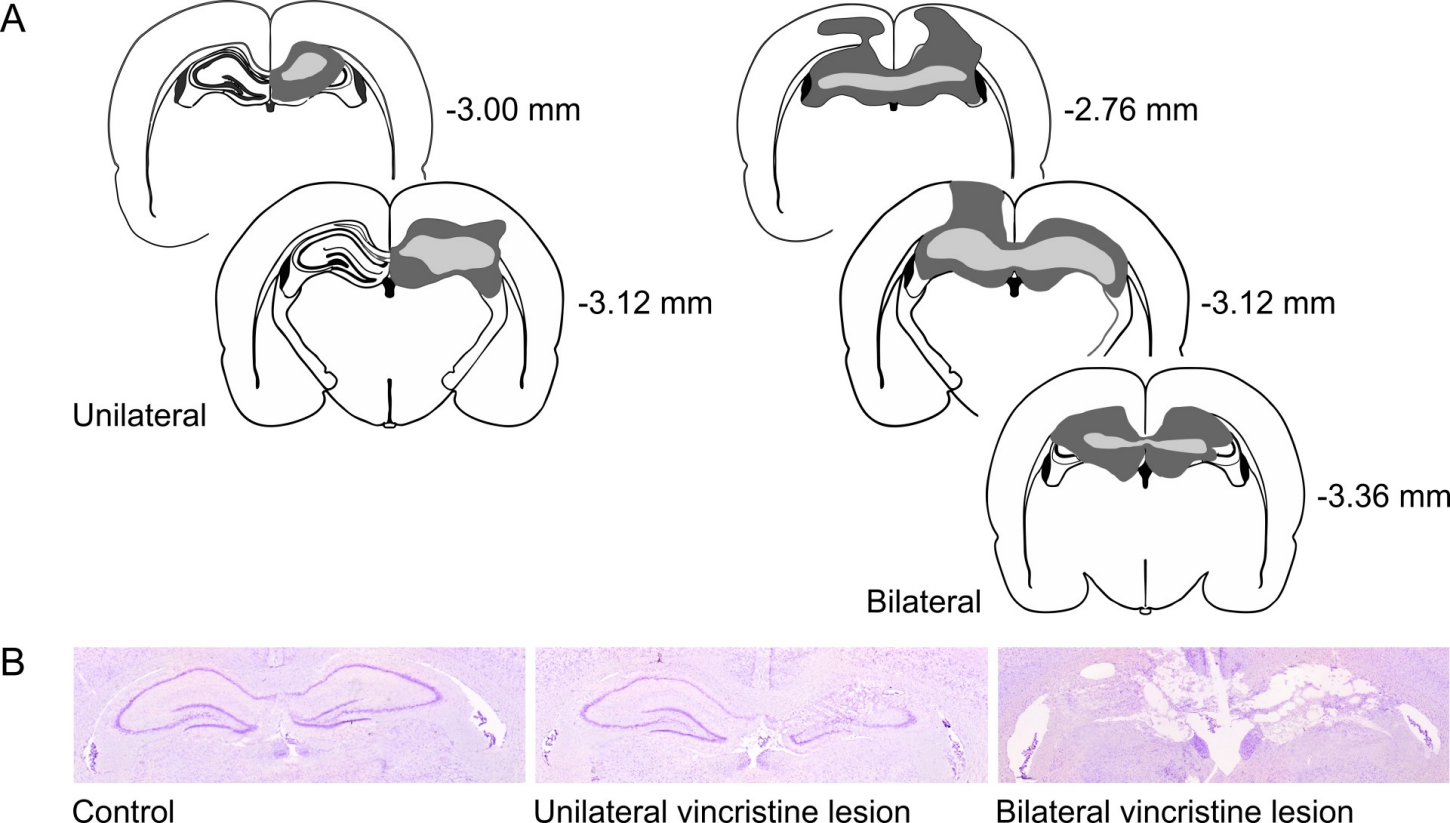
Panel A shows the extension of the smallest and largest unilateral and bilateral lesions schematically in slides based on the stereotaxic atlas by Paxinos and Watson (2004). The largest lesions are filled dark grey, the smallest lesions are filled light grey. Across all rats per lesion group, the rostro-caudal extensions of the unilateral lesions were (largest / smallest: - 2.76 mm–-3.24 mm / -3.12 mm–-3.24 mm), and for the bilateral lesions (largest/smallest: -2.04 mm–-4.36 mm / -3.00 mm–-3.12 mm). In panel B, representative histological pictures of the different groups are depicted: left: Control lesion, centre: Unilateral lesion, and right: Bilateral lesion.

Control animals (*n* = 9) presented a normal hippocampal structure (CA1, CA2, CA3 and dentate gyrus), while the treatment group presented substantial loss of the dorsal hippocampus tissue with minimal damage to the adjacent areas. Animals within the treatment group presented unilateral (*n* = 4) or bilateral lesions (*n* = 5) with one animal presenting partial bilateral lesions. Based on histology, the treatment group was divided into 2 subgroups, one containing all vincristine-injected rats with unilateral hippocampal lesions, the other containing all vincristine-injected rats with bilateral hippocampal lesions (Fig 2). One animal from the treatment group was left out of the statistical analysis due to problems with collection of the brain tissue.

### Statistical analysis

Working and reference memory, trial duration, first visit latency and inter-visit interval were averaged every 5 consecutive trials to obtain the mean of each trial block. The majority of acquisition and testing days consisted of 5 trials per day, therefore this was selected as the number of trials of choice to average for each block. Data were analyzed using the MIXED procedure in SAS (SAS, version 9.4, SAS Inst. Inc., Cary, NC) with treatment (control, unilateral and bilateral) and trial block and their interactions as fixed effects. All data were analyzed using a mixed model for repeated measures for trial blocks. Covariance structures included unstructured, compound symmetry and autoregressive order one. The structure with the lowest Schwarz’s Bayesian criterion was selected as the analysis of choice. Trial duration, first visit latency and inter-visit interval did not follow a normal distribution; therefore, these variables were log_10_-transformed. Working and reference memory were analysed untransformed. Data prior to (trial block 1–12) and after (trial block 13–20) surgery were analyzed separately. The acute effect of treatment (Vincristine lesion vs. sham lesion) was evaluated by analyzing the data from block 12 and 13 separately. The PDIFF option in SAS was used as the post-hoc test to separate the Least Square means. Post hoc tests with Bonferroni correction were performed where appropriate. Effects were considered statistically significant when *P* ≤ 0.05.

## Results

### Working memory

**Fig 3:**
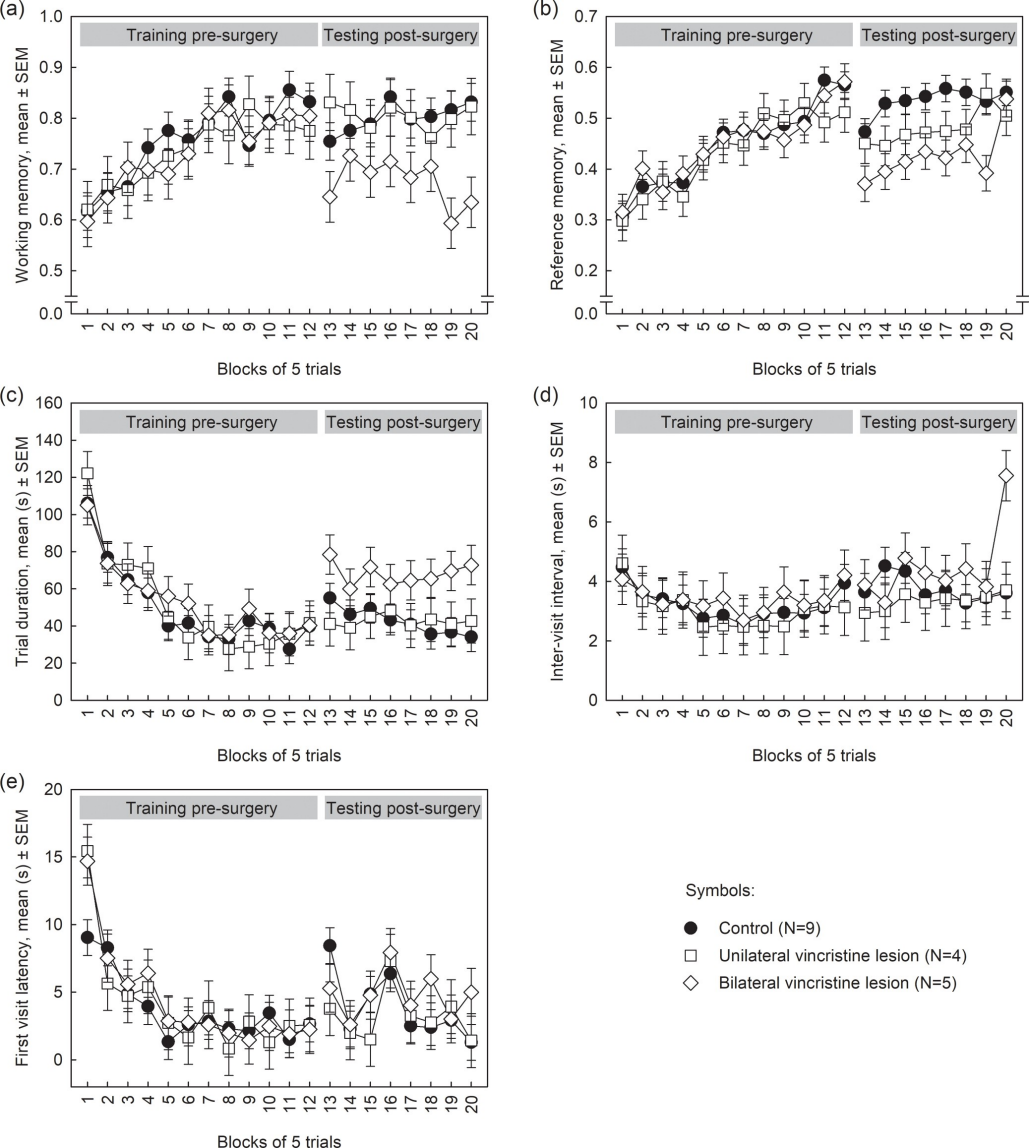
Least square means and SEMs of (a) working memory, (b) reference memory, (c) trial duration, (d) inter-visit interval and (e) first visit latency of rats of control, unilateral and bilateral hippocampal lesion groups.

Prior to surgery, the rats improved working memory performance during the course of training (F_11,165_ = 6.22, p < 0.0001) similarly in all groups (treatment effect, F_2,165_ = 0.46, p = 0.63; treatment ×trial block interaction, F_22,165_ = 0.36, p = 1.00; Fig 3A), i.e. the three groups showed similar spatial memory performance before surgery. Surgery affected overall working memory performance (F_2,105_ = 5.90, p = 0.0037): the bilateral lesion group showed a lasting lower working memory performance than the control group, but no differences were observed between both groups and the unilateral lesion group. No effects of trial block (F_7,105_ = 0.56, p = 0.79) or treatment × trial block interactions (F_14,105_ = 0.76, p = 0.71) were observed after surgery indicating that performance curves were similarly shaped.

Comparison of the last block prior to surgery and the first block after surgery showed no difference between treatments (F_2,15_ = 1.42, p = 0.2723), and trends towards an effect of trial block (F_1,15_ = 3.46, p = 0.0827) and a treatment × trial block interaction (F_2,15_ = 3.08, p = 0.0758).

### Reference memory

Prior to surgery, the rats improved reference memory performance during the course of training (F_11,165_ = 14.34, p < 0.0001) similarly in all groups (treatment effect, F_2,165_ = 0.21, p = 0.81; treatment ×trial block interaction, F_22,165_ = 0.71, p = 0.82; Fig 3B).

After surgery, reference memory performance was altered (F_2,105_ = 5.37, p = 0.0060): the bilateral lesion group had a lasting lower reference memory score than the control group, but no differences were observed between both groups and the unilateral lesion group, probably because the performance of the unilateral lesion group lay intermediate between that of the other two groups. A trial block effect (F_7,105_ = 2.49, p = 0.0210) was also observed for reference memory, indicating that post-surgery reference memory performance improved over time, similarly in all groups (treatment ×trial block interaction, F_14,105_ = 0.97, p = 0.4858).

Reference memory performance was lower (F_1,15_ = 17.38, p = 0.0008) in the trial block after surgery compared to the trial block prior to surgery. No difference between treatments was found (F_2,15_ = 1.40, p = 0.2780) and a trend towards a treatment × trial block interaction (F_2,15_ = 3.08, p = 0.0758).

### Trial duration

Prior to surgery, all groups decreased their trial duration across trial blocks (F_11,165_ = 17.70, p < 0.0001), however no treatment effect (F_2,165_ = 0.37, p = 0.69) or treatment × trial block interaction (F_22,165_ = 0.72, p = 0.82) was observed (Fig 3C). After surgery no differences were observed between treatments (F_2,105_ = 1.97, p = 0.1450), trial blocks (F_7,105_ = 1.34, p = 0.2399) or treatment × trial block interactions (F_14,105_ = 0.66, p = 0.8106).

Trial duration was longer on the trial block after surgery compared to the trial block prior to surgery (F_1,15_ = 6.38, p = 0.0233), however no difference was observed between treatments (F_2,15_ = 2.13, p = 0.1538) or treatment × trial block interaction (F_1,15_ = 1.66, p = 0.2235; Fig 3C), indicating an effect of surgery *per se*.

### Inter-visit interval

Prior to surgery, all groups decreased their inter-visit interval during the course of training (F_11,165_ = 8.60, p < 0.0001) similarly. No treatment effect (F_2,165_ = 0.73, p = 0.4813) or a treatment × trial block interaction (F_22,165_ = 0.97, p = 0.5055) was observed (Fig 3D). Vincristine injections did not affect inter-visit intervals (treatment effect, F_2,105_ = 0.13, p = 0.8798; trial block effect, F_7,105_ = 1.71, p = 0.1133; treatment × trial block interaction, F_14,105_ = 1.10, p = 0.3706; Fig 3D).

No effects of treatment (F_2,15_ = 1.64, p = 0.2267), trial block (F_1,15_ = 1.02, p = 0.3283) or a treatment × trial block interaction (F_2,15_ = 0.07, p = 0.9330) were observed between trial blocks 12 and 13.

### First visit latency

Prior to surgery, overall there was a decrease in the latency of visiting the first cone across trial blocks (F_11,165_ = 13.25, p < 0.0001). The decrease was similar in all groups (treatment effect, F_2,165_ = 0.02, p = 0.9838; treatment × trial block interaction, F_22,165_ = 1.05, p = 0.4111; Fig 3E), i.e. the rats increased their speed of starting searching for and collecting food rewards. After surgery, overall the first visit latency fluctuated across blocks, with blocks 13 and 16 having the highest latencies. No treatment (F_2,105_ = 0.17, p = 0.8479) or treatment × trail block interaction (F_14,105_ = 0.74, p = 0.7310; Fig 3E) was observed after surgery though.

First visit latency was greater on the trial block after surgery compared to the trial block prior to surgery (F_1,15_ = 9.83, p = 0.0068). However, no differences were observed between treatments (treatment effects, F_2,15_ = 0.07, p = 0.9321; treatment × trial block interaction, F_2,15_ = 0.59, p = 0.5643).

## Discussion

In the present study, bilateral hippocampal lesions induced by injection of vincristine impaired reference and working memory performance, while reference and working memory from animals with unilateral lesions did not differ from bilaterally lesioned and control rats. Reference memory, but not working memory improved with training after surgery. Thus, vincristine-induced hippocampus lesions caused cognitive deficits, but the pattern of effects observed was different for working and reference memory.

The results observed in the present study are similar to studies using other methods to create hippocampal lesions. For example, studies assessing ibotenic acid-induced lesions in the hippocampus prior to training demonstrated that animals with lesions had lower performance in a cross-shaped maze [36] and in a radial maze [37] compared to control animals. Kainic acid-induced lesions of hippocampal regions CA1 and CA3 produced deficits in the acquisition of the water maze task [38]. Similar studies have reported impaired reference and working memory in animals with hippocampal lesions caused by electrolysis 1 month post-surgery, and impaired reference memory 6 months post-surgery [39]. As such, using vincristine offers the opportunity to extend the repertoire of compounds for lesioning hippocampal areas beyond the use of excitotoxins (e.g. ibotenic acid or kainic acid), or electrolytic lesioning [36–39]. Pending further validation, rodents with vincristine induced lesions of (subfields) of the hippocampal formation may be considered as an alternative for animal models of cognitive deficits. The present study and earlier studies using the Morris water maze task [9] have shown that these lesions create animals with compromised spatial discrimination performance.

Spatial learning deficits have been previously reported when assessing the effect of different volumes and concentrations of vincristine injected bilaterally into the dorsal hippocampus [9]. Interestingly, Eijkenboom and van der Staay [9] reported that bilateral lesions induced by 1.0 μl vincristine at a concentration of 0.18 mg/ml (i.e., the volume and concentration used in the present study), did not impair acquisition and retention of Wistar rats tested in the MWM. A likely explanation for the differences observed between studies is the difference in timing of treatment and the complexity of the spatial orientation task used. In the study by Eijkenboom and van der Staay [9], vincristine was injected before the start of training, whereas vincristine in our study was injected after the animals had acquired the task. Furthermore, the conefield task may be more sensitive to disruption of the functions of the hippocampal formation than the MWM, perhaps because of its greater complexity.

In the present study, animals were first trained in the task, then received the chemotherapeutic drug and shortly after were tested. This is different from the majority of research in this field, in which chemotherapeutic drug treatments are typically applied before training [40–47]. Importantly, fear conditioning studies have reported differences in the effects of hippocampal lesions when performed prior to or after training [48,49]. Likewise, differences in the formation of spatial memory in rats that were trained in a complex environment prior to and after receiving NMDA-induced excitotoxic hippocampal lesions have been reported [50]. Thus, animals that were trained for as little as 2 weeks prior to hippocampal lesions, developed spatial memories which were resistant to hippocampal lesions, while animals that received training 3 months after hippocampal lesions, had a similarly impaired performance than animals with hippocampal lesions with no previous training, suggesting the importance of an intact hippocampus for developing a spatial representation of the environment. Similar to a previous study [50], rats in the present study were trained for a short period (13 days) prior to hippocampal lesions. Although animals in the previous study [50] had good memory performance, challenges were observed when the task required flexible use of existing representations. For example, if a route was blocked, animals with hippocampal lesions took a longer way to reach the desired location compared to control rats.

Previous studies have shown that hippocampal lesions do not affect contextual fear conditioning when animals are trained prior to lesions, suggesting that although context learning requires the hippocampus, context representations also exist outside the hippocampus [51]. Intact anterograde spatial memory has also been reported in rats trained in an open field task, prior to hippocampal damage [52]. This may explain why the speed of reference memory re-acquisition seems not to be affected by the surgery in the present study, whereas lesioning induced a generally lower level of reference memory performance. Thus training prior to hippocampal lesions could have created spatial representations outside the hippocampus, such as in the anterior thalamic nuclei [53] or the hippocampal-diencephalic-cingulate network [54], which compensates for the loss caused by the dorsal hippocampal lesions. Importantly, the prefrontal cortex and the hippocampus have been reported to be involved in spatial working memory encoding, but they are not critical structures for the maintenance or retrieval of spatial cues [55]. However, working memory during spatial navigation updating is highly dependent on the hippocampus [56], consistent with our finding that a bilateral hippocampal lesion had a lasting effect on spatial working memory. Testing the animals for a longer period of time will be needed to clarify if working memory as well as reference memory were permanently or transiently impaired by the damage of the hippocampal cells produced by vincristine.

Vincristine has been reported to interrupt the microtubule dynamics through the inhibition of the expression of tubulin and fibronectin and the downregulation of the gene matrix metalloproteinase 10 [8] which leads to mitotic arrest and apoptosis [4–6]. Cells in the G_1_ phase, are then susceptible to vincristine, which causes cell death. However, cells in the late G_1_ phase and early S-phase are unaffected until mitosis, when they will die due to mitotic arrest [57]. The effect of vincristine on the microtubule dynamics has been reported to affect axonal transport in the nervous system [58], which has been associated with an axon degeneration program consisting of a decrease of axonal nicotinamide mononucleotide adenylyltransferase 2 (NMNAT2) levels, activation of sterile alpha and toll/interleukin-1 receptor motif-containing 1 (SARM1) and depletion of NAD^+^ followed by axon fragmentation [59]. Persistent working memory, and transient reference memory deficits observed in this study, are therefore likely due to the dorsal hippocampus axon deterioration. Importantly, chemotherapeutic drugs that share the same mechanism of action, can have a different effect in the CNS. For example, colchicine and vincristine cause cell death by disrupting microtubule formation, but colchicine selectively damages dentate granule cells with minimal damage to hippocampal pyramidal cells, while vincristine damages both types of cells [60]. Spatial learning deficits have been reported in rats tested in the MWM, after bilateral dentate gyrus injections of colchicine, which selectively damages dentate granule cells [61]. Therefore, it is not surprising to observe spatial deficits after bilateral injections of vincristine, which damages all cell types in the target brain area and adjacent brain structures.

Histological analyses revealed variable lesion sizes within the treatment group; four out of nine animals of the treatment group presented unilateral instead of bilateral lesions. From the unilateral lesion group (n = 4), two animals presented total dorsal hippocampus lesions while two presented partial lesions. In the bilateral lesion group (n = 5) four animals presented total dorsal hippocampus lesions while one animal presented a partial dorsal hippocampus lesion. The reason for these variable lesions is unclear, but could include practical issues, such as problems with vincristine infusions, or relative resistance of these rats to vincristine-induced neurotoxicity. Based on our results we could hypothesize that a unilateral intact hippocampus can compensate for the loss of the affected hippocampal structure as no differences were observed between unilateral and control animals. However, no differences were observed between the unilateral and the bilateral lesion group to support this hypothesis, likely because the performance of the unilateral lesion group lay intermediate between that of the other two groups (control and bilaterally lesioned group). Inspection of Fig 3B suggests that unilateral lesions may have impaired reference memory performance slightly. Caution should therefore be taken when interpreting these results as the number of animals in the unilateral and bilateral lesion group is small.

After surgery, there were no group differences or trial block effects for trial duration, inter-visit interval and first visit latency, meaning that animals did not have problems executing the task but that the problem was limited to memory deficits. Thus, the motivation to seek for food rewards was not affected, which is likely due to the fact that relevant brain structures involved in food intake such as the forebrain, hypothalamic and hindbrain circuits [62] as well as the nucleus accumbens [63,64] were spared by the vincristine lesions.

A comparison of block 12 (last trial block before surgery) and block 13 (first trial block after surgery) was done to assess if surgery affected acute retention of task performance. There were no block effects for working memory or inter-visit interval, however a trial block effect showed that rats had a lower reference memory performance, a greater trial duration and a greater first visit latency after lesioning (i.e. on trial block 13). The results observed for reference memory, trial duration and first visit latency could be due to the recovery time in which rats were not trained in the task. However, no signs of forgetting have been found in the cone-field task in a previous study after a retention interval of four months [65]. Therefore, it is unlikely that the short period between the end of training and retention testing impaired memory.

Limitations of the present study that need to be considered include a small sample size, lesions extending to cortical areas outside of the dorsal hippocampus, and that in practice, humans do not receive intra-hippocampal vincristine. It would be interesting to repeat this same experimental procedure with a long-term systemic vincristine treatment, as it is common in clinical practice. Based on the previously mentioned literature [40–47] we would expect to see cognitive deficits after systemic vincristine treatment, but by training the animals before treatment we could see if chemotherapy affects a previously learned task or if it only affects the acquisition of new information. Of note, a previous study has shown that chemotherapy not only affects short term memory but also tasks that patients had learned before chemotherapy treatment, such as paying the bills or driving to a known place [66].

## Conclusion

In the present study, we found that bilateral dorsal hippocampus lesions induced by vincristine caused a decrease in performance in working and reference memory in the cone field spatial discrimination task. Together with previous findings [9] this suggests that cytostatics reaching the brain can induce severe neuronal and cognitive damage. Future studies are needed to elucidate whether systemic chemotherapeutic treatment can result in, for example, blood-brain-barrier deficits or inflammatory processes that allow cytostatic drugs to reach the brain during the treatment of cancer.

## Supporting information

S1 Data (XLSX)
